# Long-Term Cardiovascular, Thromboembolic, and Malignancy Safety of Janus Kinase (JAK) Inhibitors in Dermatologic and Rheumatologic Populations: A Narrative Comparative Review

**DOI:** 10.7759/cureus.116911

**Published:** 2026-09-25

**Authors:** Shakira Bou Rolon, Andrea N Ortiz Echevarria, Monica Burgos Claudio, Edgar A Solis Esquilin, Pedro J Rodriguez Ramirez

**Affiliations:** 1 Dermatology, Ponce Health Sciences University, Puerto Rico, USA; 2 Dermatology, Universidad Central del Caribe School of Medicine, Puerto Rico, USA; 3 Internal Medicine, Hospital Universitario Ramon Ruiz Arnau, Puerto Rico, USA

**Keywords:** alopecia areata, atopic dermatitis, cardiovascular safety, janus kinase inhibitors (jak inhibitors), major adverse cardiovascular events (mace), malignancy, psoriatic-arthritis, rheumatoid arthritis, venous thromboembolism (vte)

## Abstract

Janus kinase inhibitors are increasingly used in dermatology and rheumatology, but concerns about cardiovascular events, thromboembolism, and malignancy continue to influence long-term treatment decisions. This narrative comparative review examines evidence from randomized trials, integrated safety analyses, long-term extensions, selected observational studies, and regulatory sources, focusing on rheumatoid arthritis, psoriatic arthritis, atopic dermatitis, and alopecia areata. Topical agents and selective tyrosine kinase 2 inhibition are considered separately because of important differences in systemic exposure and pharmacology. The original, informally assembled evidence base was supplemented by a targeted literature update. ORAL Surveillance identified an important safety signal for tofacitinib compared with tumor necrosis factor inhibitors in patients with rheumatoid arthritis and cardiovascular risk factors. Long-term dermatology studies have generally reported low absolute event rates, although younger study populations, restrictive eligibility criteria, limited long-term comparator data, and treatment discontinuation affect how these findings should be interpreted. Observational studies in dermatology have produced mixed results, including thromboembolic associations that warrant further investigation. Psoriatic arthritis provides additional evidence across the clinical boundaries of both specialties. Differences in patient populations, study design, outcome definitions, and statistical measures limit direct comparisons across studies. Treatment decisions should follow indication-specific prescribing requirements and consider cardiovascular disease, prior thrombosis, smoking, age, malignancy history, and available alternatives. Additional long-term comparative studies are needed to better distinguish patient-, disease-, and treatment-related risks.

## Introduction and background

Janus kinase (JAK) inhibitors have expanded treatment options for immune-mediated inflammatory disease. Their oral administration and effects on multiple cytokine pathways are clinically useful in rheumatoid arthritis (RA), psoriatic arthritis (PsA), atopic dermatitis (AD), and alopecia areata (AA). However, sustained treatment requires consideration of uncommon but serious outcomes that may not be adequately characterized in short efficacy trials.

The principal outcomes examined here are major adverse cardiovascular events (MACE), venous thromboembolism (VTE), and malignancy. Three-point MACE generally comprises cardiovascular death, non-fatal myocardial infarction, and non-fatal stroke. VTE includes deep vein thrombosis (DVT) and pulmonary embolism (PE). Malignancy excluding non-melanoma skin cancer (NMSC) is distinguished from NMSC throughout this review. Individual studies may differ in event definitions, adjudication, surveillance, and follow-up windows; similarly named endpoints should therefore not automatically be assumed equivalent. ORAL Surveillance used adjudicated cardiovascular and malignancy outcomes and provides the central randomized safety comparison discussed below [1].

ORAL Surveillance evaluated tofacitinib against tumor necrosis factor inhibitors (TNFis) in an older RA population with additional cardiovascular risk. Its findings prompted updated US Food and Drug Administration (FDA) warnings and European Medicines Agency (EMA) risk-minimization measures [2,3]. Applying these findings to dermatology requires careful attention to the population, drug, dose, formulation, and comparator. A younger trial population can have a lower absolute incidence of serious events without demonstrating a smaller treatment-associated relative risk.

This narrative review compares the strengths and limitations of the evidence across specialties. It primarily addresses systemic JAK inhibition in RA, PsA, AD, and AA. Topical ruxolitinib and delgocitinib are considered separately because the route of administration changes exposure and regulatory interpretation. Deucravacitinib is discussed as a pharmacologically distinct allosteric tyrosine kinase 2 (TYK2) inhibitor, rather than pooled with conventional JAK1/2/3 inhibitors. The purpose is clinical interpretation, not a statistical ranking of agents or an exhaustive review of every indication.

## Review

Methods

This manuscript was based on references gathered informally from July to September 2026. Searches focused on PubMed-indexed publications, PubMed Central articles, publisher-hosted reports, and FDA and EMA documents. Because this was a targeted narrative update rather than a systematic review, we did not conduct an exhaustive search through the native interfaces of multiple bibliographic databases. We combined drug names, disease names, trial program names, and terms related to cardiovascular events, thromboembolism, malignancy, long-term safety, and observational evidence in iterative searches.

We considered English-language reports available by the update date without a prespecified lower publication date limit. Study selection was purposive, with priority given to randomized comparative studies, longer integrated safety analyses and extensions, observational studies with active comparators, regulatory documents, and mechanistic studies relevant to the review questions. Shorter comparative trials were included when they helped clarify the contribution or limitations of the evidence. Existing systematic reviews were used for context rather than counted as additional patient cohorts. When reports overlapped, we identified the relevant data cut and study purpose and did not combine sample sizes or exposure across overlapping analyses. Regulatory and pharmacokinetic information was used to inform the separate discussion of topical therapy.

Evidence was summarized qualitatively according to disease, agent, formulation, and study design. When available, we retained the reported denominators, duration, comparators, event definitions, confidence intervals (CIs), and analysis populations. We did not perform a new meta-analysis, indirect treatment comparison, standardized cross-study risk estimate, or formal risk-of-bias assessment. Instead, limitations related to selection, attrition, confounding, sponsorship, and outcome ascertainment were considered narratively. The review did not use a registered protocol, duplicate independent screening, or a PRISMA study-flow count. These features limit reproducibility and completeness and should be considered when interpreting this targeted narrative synthesis.

The JAK-signal transducer and activator of transcription pathway and biological plausibility

JAK1, JAK2, JAK3, and TYK2 connect cytokine receptors with signal transducer and activator of transcription (STAT) proteins. After receptor stimulation, STAT proteins are phosphorylated and translocate to the nucleus, where they alter gene expression. Because JAK inhibition can affect inflammation, hematopoiesis, and immune function at the same time, clinical monitoring should be tailored to the individual agent rather than assuming identical effects across the class [4].

Drug selectivity is relative and varies with concentration, cell type, and the cytokine assay being studied. Tofacitinib is commonly described as predominantly JAK1/3-directed, baricitinib as JAK1/2-directed, and upadacitinib and abrocitinib as JAK1-preferential. Studies in human leukocyte populations nevertheless show substantial overlap in cytokine signaling effects, illustrating why these labels should not be interpreted as complete pathway specificity. Common gamma-chain cytokines such as IL-2 and IL-15 signal through JAK1/JAK3, whereas interferon-gamma receptor signaling involves JAK1/JAK2. Therefore, inhibition of interferon-gamma signaling should not be attributed specifically to JAK3 [5].

JAK2 participates in erythropoietin and thrombopoietin signaling and therefore has an important role in erythroid and megakaryocytic biology. Changes in blood-cell production and platelet signaling are biologically relevant, but their overall effect on thrombosis cannot be predicted from kinase inhibition alone. In experimental human platelets, ruxolitinib and baricitinib impaired glycoprotein-VI-mediated responses rather than producing a consistent proaggregatory effect. These findings make it difficult to attribute clinical VTE to a simple mechanism involving disrupted thrombopoietin signaling. Background inflammation, vascular risk, treatment effects, and the coagulation environment may all contribute [4,6].

Immune surveillance provides another potential link between JAK inhibition and malignancy. In preclinical breast cancer models, JAK inhibition reduced natural killer cell-mediated antitumor activity and increased metastasis. This finding supports biological plausibility but does not establish the mechanism or magnitude of cancer risk in patients treated for inflammatory disease. Tumor cell signaling and host immune effects may also act in different directions. Figure 1 therefore separates established signaling relationships from proposed links to clinical outcomes [7].

**Figure 1:**
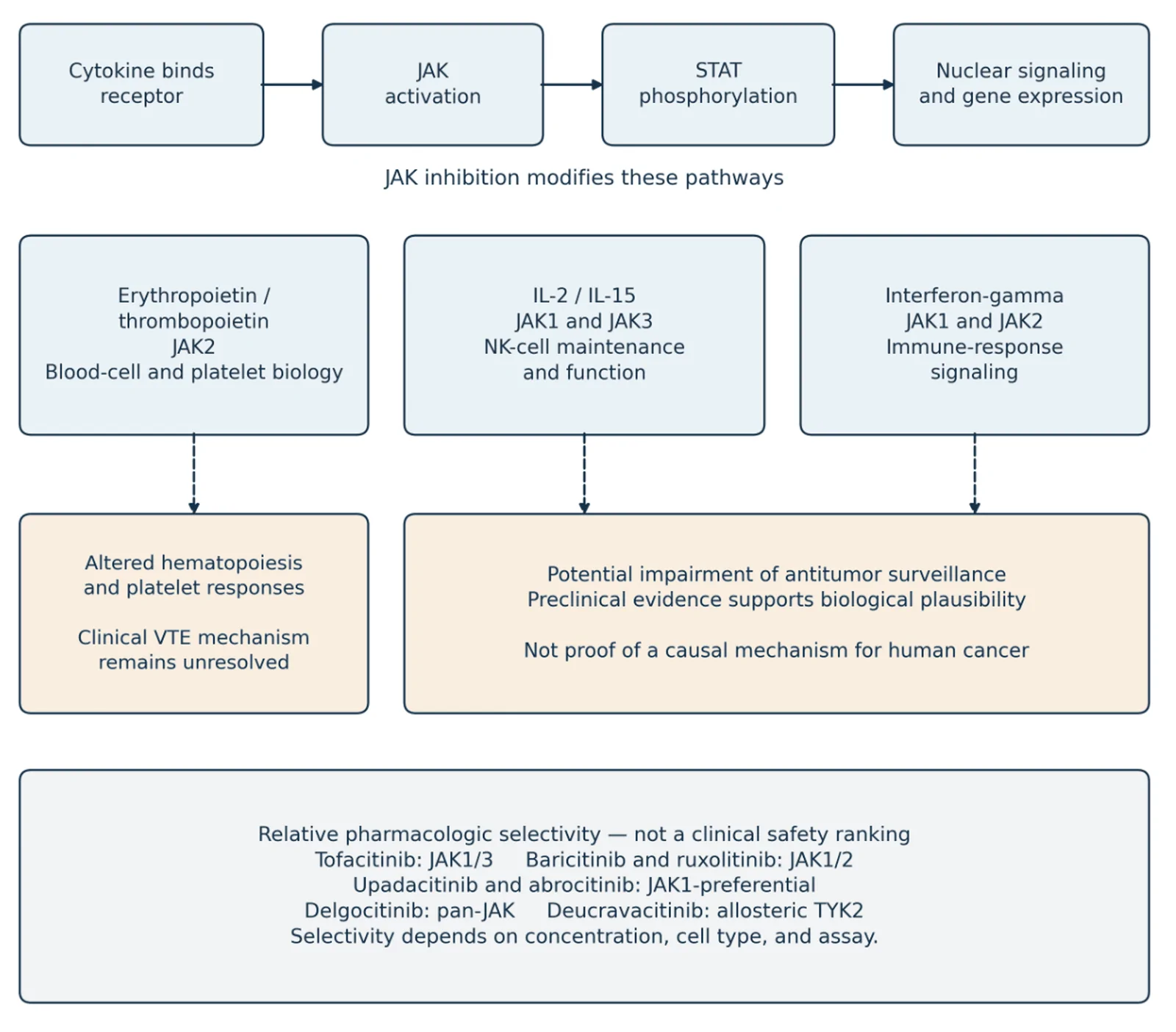
JAK signaling and potential safety pathways JAK signaling, relative drug selectivity, and potential adverse-outcome pathways. Solid arrows show the general signaling sequence; dashed arrows indicate proposed links whose clinical significance is not established. The diagram does not imply that JAK2 inhibition uniformly increases platelet activation or that in vitro selectivity predicts clinical safety. The original author-proposed schematic is informed by the cited pharmacology, experimental, and regulatory sources [4-7]. IL, interleukin; JAK, Janus kinase; STAT, signal transducer and activator of transcription; TYK2, tyrosine kinase 2; VTE, venous thromboembolism.

Randomized safety evidence in RA

ORAL Surveillance enrolled 4,362 patients aged 50 years or older with active RA despite methotrexate and at least one additional cardiovascular risk factor. Participants received tofacitinib 5 mg twice daily, tofacitinib 10 mg twice daily, or a TNFi. Over a median follow-up of four years, MACE occurred in 3.4% of patients receiving combined tofacitinib doses and 2.5% receiving TNFi (hazard ratio (HR), 1.33; 95% CI, 0.91-1.94). Malignancy excluding NMSC occurred in 4.2% and 2.9%, respectively (HR, 1.48; 95% CI, 1.04-2.09). The prespecified non-inferiority criterion was not met for either endpoint. Importantly, the MACE CI crossed 1.0; this is different from failure to demonstrate non-inferiority, and the two findings should not be interpreted as showing equivalence [1].

The higher-dose tofacitinib arm was modified after concerns emerged regarding PE and mortality. In its final evaluation, the FDA also identified increased thrombosis and mortality concerns with the lower dose. Although ORAL Surveillance enrolled a population already at elevated baseline risk, that does not negate the randomized comparison within the population studied. The main limitation is how far those results can be generalized to different patient populations and other JAK inhibitors [2].

Post hoc analyses provide additional information about where risk may be concentrated. Among participants with established atherosclerotic cardiovascular disease (ASCVD), MACE occurred in 8.3% and 7.7% of patients receiving the two tofacitinib doses, compared with 4.2% receiving TNFi. Other analyses identified older age and smoking history as markers associated with higher risk. These subgroup findings are useful for clinical discussion, although they cannot establish that younger patients who have never smoked are free of treatment-associated risk. The malignancy analysis similarly found higher event incidence among patients with greater baseline cardiovascular risk and smoking-related characteristics [8-10].

Broader rheumatology programs and PA

Evidence beyond tofacitinib is important when considering whether safety findings extend across the JAK inhibitor class. RA-BEACON and RA-BUILD evaluated baricitinib in different treatment-experienced RA populations and provided randomized, placebo-controlled efficacy and safety data. However, their approximately 24-week controlled periods were not designed as event-driven, long-term comparative safety trials [11,12]. A later integrated baricitinib analysis included 3,770 exposed patients and 14,744.4 patient-years (PY), with a median exposure of 4.6 years and a maximum of 9.3 years. Reported incidence rates per 100 PY were 0.5 for MACE, 0.5 for DVT/PE, and 0.9 for malignancy excluding NMSC. These data extend the duration of observation, but the long-term rates were not supported by a concurrent comparator throughout follow-up [13].

The upadacitinib SELECT program is represented by a 2025 integrated analysis of 8,632 treated patients and 27,164.2 PY across several indications, including 3,209 patients with RA and 907 with PsA. Adalimumab and methotrexate reference groups were available in selected programs. In RA and PsA, MACE, VTE, and malignancy excluding NMSC occurred at rates that were numerically similar to those in the active-comparator groups, while NMSC was more frequent with upadacitinib. These integrated comparisons are descriptive and were not designed to establish equivalence or to show that upadacitinib carries a lower serious-event risk than tofacitinib [14].

PsA is particularly relevant because it spans both dermatologic and rheumatologic practice and illustrates why specialty alone is an imperfect way to judge risk. OPAL Balance followed 686 tofacitinib-treated patients with PsA for up to 48 months. Reported rates per 100 person-years were 0.2 for MACE, 0.1 for PE, and 0.7 for malignancy excluding NMSC. Because the extension was open-label and lacked a sustained randomized active comparator, these findings are best viewed as complementary long-term data. In addition, the reported PE rate should not be treated as equivalent to a combined DVT/PE endpoint [15].

Comparative and long-term evidence in dermatology

Dermatology trials often enroll younger patients and record relatively few serious cardiovascular or malignant events. A meta-analysis of 35 randomized trials involving 20,651 participants found no statistically significant increase in a composite of MACE and all-cause mortality or in VTE. However, mean follow-up was only about 4.9 months, which limits the ability to evaluate uncommon or delayed adverse outcomes. The study's combined MACE/mortality endpoint should also not be interpreted as conventional three-point MACE [16].

Active-comparator evidence is also available in dermatology. JADE COMPARE randomized 838 adults with AD to abrocitinib, dupilumab, or placebo in addition to background topical therapy. The 16-week study provides useful comparative information on short-term efficacy and tolerability, but its duration and small number of serious events are insufficient to define long-term comparative risks of MACE, VTE, or malignancy. Its findings are therefore most informative when considered alongside longer extension studies and observational data [17].

Upadacitinib in AD

The six-year integrated analysis of Measure Up 1, Measure Up 2, and AD Up included 2,683 patients: 1,337 receiving upadacitinib 15 mg and 1,346 receiving 30 mg, corresponding to 4,435.2 and 4,752.5 PY of exposure. Reported MACE rates were 0.2 and less than 0.1 per 100 PY, respectively, while VTE occurred at a rate of 0.1 per 100 PY with either dose. Malignancy rates, excluding NMSC, were 0.3 and 0.5 per 100 PY. Because the placebo-controlled phase did not continue throughout the six years, these long-term data describe observed event rates rather than a six-year randomized comparison with placebo or biologic therapy [18].

A published correction is important when interpreting this report. It clarified that 11 VTE cases occurred, six were serious adverse events, and none were fatal; it also corrected the one-year discontinuation rates. The low event rates observed over long-term follow-up are reassuring descriptively, but differences between dose groups and the absence of an increasing cumulative rate are not sufficient to establish a dose-related safety advantage or rule out delayed harm [19].

Baricitinib in AD and AA

The final BREEZE-AD integrated analysis included 2,637 adults with 5,216.2 PY of exposure, a median exposure of 1.6 years, and a maximum of 4.6 years. Reported incidence rates per 100 PY were 0.19 for MACE, 0.07 for DVT/PE, and 0.39 for malignancy excluding NMSC. The maximum follow-up represents the longest observed exposure rather than the experience of every participant. Comparisons with external background rates can provide context but do not offer the same control for confounding as an internal randomized comparator [20].

The BRAVE-AA analysis included 1,303 patients and 2,789.7 PY, with a median exposure of 2.3 years and a maximum of four years. Reported rates per 100 PY were less than 0.1 for MACE, 0.1 for VTE, and 0.2 for malignancy excluding NMSC. The trials excluded patients with several important cardiovascular and thrombotic risk factors. As a result, these low event rates may not fully represent patients with prior VTE or substantial comorbidity who would be encountered in routine practice [21].

Abrocitinib in AD

The integrated abrocitinib safety update included 3,802 patients with 5,213.9 PY of exposure and follow-up approaching four years. Age-stratified results were reported from the consistent-dose cohort rather than the entire integrated population. In that cohort, malignancy excluding NMSC occurred at 2.4 per 100 PY (95% CI, 0.6-6.0) among patients aged 65 years or older and 0.1 (0.0-0.4) among adults aged 18 to less than 65 years. Corresponding VTE rates were 1.7 (0.4-5.1) and 0.1 (0.0-0.3). The wide CIs, particularly in older patients, reflect limited precision. These within-treatment subgroup findings describe observed age-related differences but do not establish age-specific causal effects compared with an untreated population [22].

Topical agents and systemic exposure

Topical JAK inhibitors deserve separate consideration because systemic exposure differs substantially from oral therapy. In phase II/III pharmacokinetic studies of AD, ruxolitinib 1.5% cream used twice daily had a mean systemic bioavailability of approximately 6.22%, although exposure varied with treatment characteristics. This lower exposure supports evaluating topical and oral formulations separately, but it does not mean systemic absorption is absent [23]. The US label for ruxolitinib cream includes boxed warnings for serious infections, mortality, malignancy, MACE, and thrombosis based in part on experience with oral JAK inhibition. It also sets indication-specific limits on treated body surface area and quantity used [24].

In the integrated TRuE-V vitiligo program, 637 patients received ruxolitinib cream, accounting for 867.9 PY of exposure over 104 weeks. No MACE or deaths were reported, and malignancy and thromboembolic events were uncommon and were not considered treatment-related by investigators. However, vehicle and ruxolitinib exposure periods differed, and some participants contributed to both treatment categories. The study therefore does not represent a uniform two-year randomized comparison. The rarity of these events and the duration of follow-up also leave uncertainty about small long-term risks [25].

Delgocitinib should be considered on its own rather than assumed to share the same indications or labeling as ruxolitinib. The US label for delgocitinib cream covers moderate-to-severe chronic hand eczema in adults who have had an inadequate response to topical corticosteroids or cannot use them. Its precautions address infections, NMSC, immunization, and potential JAK-related risks, but the label does not use the same boxed-warning presentation as ruxolitinib cream. These differences highlight the importance of describing each topical agent according to its specific indication, formulation, and jurisdiction [26].

Selective TYK2 inhibition

Deucravacitinib binds the regulatory domain of TYK2 and is therefore considered separately from conventional JAK1/2/3 inhibitors. In the four-year POETYK PSO analysis, 1,519 exposed patients contributed 4,392.8 person-years, with reported MACE and VTE rates of 0.32 and 0.07 per 100 person-years, respectively [27]. A subsequent five-year report included 5,046.7 person-years and reported rates of 0.34 for MACE, 0.06 for VTE, and 0.51 for malignancy excluding NMSC per 100 person-years. These long-term findings are useful for describing observed safety but do not provide a direct comparative estimate against other agents [28].

Observational evidence and its interpretation

Real-world studies add another perspective, although their findings are not entirely consistent. Safety of TofAcitinib in Routine care patients with Rheumatoid Arthritis (STAR-RA) compared tofacitinib with TNFi using US claims data. For the composite of myocardial infarction and stroke, the pooled weighted HR was 1.01 (95% CI, 0.83-1.23) in the routine-care cohort and 1.24 (0.90-1.69) in a cohort designed to resemble ORAL Surveillance eligibility. Neither CI excluded the null. Differences in eligibility criteria and outcome definitions mean that these estimates are not directly interchangeable with the randomized three-point MACE findings from ORAL Surveillance [29]. A separate STAR-RA analysis did not detect an increase in overall malignancy, although limited follow-up and residual confounding make delayed effects difficult to exclude [30].

A Swedish registry study reported a different signal for thromboembolism. In patients with RA, JAK inhibitor use was associated with a higher VTE incidence than TNFi use (adjusted HR, 1.73; 95% CI, 1.24-2.42), driven mainly by PE. This finding should be considered alongside studies that did not detect cardiovascular differences. Variation in outcomes, prescribing patterns, follow-up, and baseline patient risk can contribute to apparently different results across observational datasets [31].

Observational evidence in dermatology is similarly mixed. Kridin and colleagues examined three-year outcomes in propensity-matched AD cohorts and found that JAK inhibitor treatment, compared with dupilumab, was associated with PE (HR, 2.75; 95% CI, 1.19-6.38) and DVT (HR, 2.54; 95% CI, 1.14-5.64), while myocardial infarction and stroke were not significantly increased. These associations do not establish causality and may be influenced by residual confounding or treatment channeling [32]. By contrast, Zhao and colleagues matched 1,978 JAK inhibitor initiators with 1,978 IL-4/IL-13 inhibitor initiators and found no evidence of increased cardiovascular or cancer risk. That analysis was limited by the number of events and by imperfect ascertainment of follow-up and treatment discontinuation [33].

Both dermatology studies used the TriNetX network, so their populations may overlap and should not automatically be treated as fully independent. They also evaluated different outcomes: a study finding no increase in cardiovascular disease or cancer does not directly address a separate VTE association. As with other database studies, information on smoking, disease severity, adherence, treatment duration, and outcome adjudication may be incomplete. Propensity matching can balance measured characteristics but cannot account for unmeasured confounding. Even with these limitations, these studies broaden the evidence beyond sponsored extension programs and highlight questions that require prospective confirmation.

Comparing absolute risk without ranking treatments

A patient's baseline risk strongly influences how a relative treatment effect translates into absolute harm. Age, ASCVD, smoking, obesity, and previous thrombosis can vary substantially both between specialties and among patients with the same dermatologic diagnosis. Patients with RA or PsA may also have different histories of chronic inflammation, glucocorticoid exposure, and other immunomodulatory treatment. These differences complicate comparisons between populations, although they do not diminish the value of randomized comparisons within a study.

Comparator choice also affects interpretation. A meta-analysis across indications found that malignancy results differed depending on whether JAK inhibitors were compared with TNFi, placebo, or methotrexate. Therefore, the absence of a signal against placebo should not be assumed to imply the same relationship against every active comparator [34]. Statistical measures also need to be kept distinct: an exposure-adjusted incidence rate describes events during observed follow-up, whereas an HR estimates relative event hazard against a specific comparator. Percentages, events per 100 PY, patients with an event per 100 PY, and HRs are not interchangeable. Tables 1, 2 separate study design from outcome summaries to make these differences clearer.

**Table 1:** Study populations and design features of selected systemic JAK safety datasets The rows are selected datasets, not an exhaustive trial inventory. Exposed populations may include participants who switched treatment; denominators are not necessarily original randomized allocations. PY denotes patient-years. Maximum follow-up does not represent typical exposure. Overlapping programs are not independent samples and must not be summed. AA, alopecia areata; AD, atopic dermatitis; JAK, Janus kinase; PsA, psoriatic arthritis; RA, rheumatoid arthritis; TNFi, tumor necrosis factor inhibitor.

Study and population	Sample (N)	Design	Comparator	Follow-up
ORAL Surveillance RA [1]	4,362	Open-label randomized safety trial; adjudicated endpoints	TNFi versus tofacitinib 5 or 10 mg twice daily	Median 4 years
Baricitinib RA integrated program includes RA-BEACON/RA-BUILD [13]	3,770 exposed	Integrated trials and long-term extension	No sustained concurrent control for all-bari long-term rates	14,744.4 PY; median 4.6, maximum 9.3 years
Upadacitinib SELECT RA/PsA subset [14]	RA: 3,209; PsA: 907 exposed	Descriptive integrated phase 3 programs	Selected RA/PsA trials: adalimumab; RA also methotrexate	Duration varies by program; RA median: 216.3 weeks
OPAL Balance PsA [15]	686 exposed	Open-label extension with withdrawal sub-study	No sustained active comparator for extension rates	Up to 48 months
Measure Up 1/2 and AD Up AD [18,19]	2,683 exposed	Integrated phase 3 trials and extensions	Initial placebo phase; no placebo/biologic comparator throughout 6 years	9,187.7 PY; up to 6 years
BREEZE-AD integrated program AD [20]	2,637 exposed	Eight-trial integrated analysis	No sustained concurrent control for all-bari long-term rates	5,216.2 PY; median 1.6, maximum 4.6 years
BRAVE-AA1/2 AA [21]	1,303 exposed	Integrated trials with extensions and allocation changes	Placebo in parent trials; long-term all-bari cohort is descriptive	2,789.7 PY; median 2.3, maximum 4 years
Abrocitinib integrated program AD [22]	3,802 overall	Integrated trials and extension; separate consistent-dose analyses	Long-term age strata do not constitute a randomized comparison	5,213.9 PY; up to approximately 4 years

**Table 2:** Outcome summaries for the datasets in Table 1 All incidence rates are source-reported per 100 PY; parentheses contain 95% confidence intervals where reproduced. ORAL Surveillance reports proportions and HRs for combined tofacitinib doses versus TNFi, not incidence rates in these cells. An IR is not equivalent to an HR. Definitions and at-risk windows differ across reports; no standardized or adjusted between-study comparison was calculated. The abrocitinib age strata belong to a consistent-dose subgroup, not all 3,802 participants. CI omissions do not imply certainty. These data must not be used to rank the safety of agents or specialties. AA, alopecia areata; AD, atopic dermatitis; DVT, deep vein thrombosis; FDA, Food and Drug Administration; MACE, major adverse cardiovascular event; NMSC, non-melanoma skin cancer; PE, pulmonary embolism; PsA, psoriatic arthritis; RA, rheumatoid arthritis; TNFi, tumor necrosis factor inhibitor; VTE, venous thromboembolism.

Dataset	MACE	Thromboembolism	Malignancy excluding NMSC unless stated
ORAL Surveillance [1]	3.4% vs 2.5%; HR: 1.33 (95% CI: 0.91-1.94)	Dose-related concerns; see text and FDA evaluation	4.2% vs 2.9%; HR: 1.48 (1.04-2.09)
Baricitinib RA [13]	IR: 0.5	DVT/PE IR: 0.5	IR: 0.9
Upadacitinib RA/PsA [14]	Numerically similar to reference comparators	Numerically similar to reference comparators	Numerically similar excluding NMSC; NMSC more frequent
OPAL Balance [15]	IR: 0.2 (0.1-0.6)	PE IR: 0.1 (0.0-0.3); not a combined VTE estimate	IR: 0.7 (0.4-1.2)
Upadacitinib AD [18,19]	15 mg: 0.2 (0.1-0.3); 30 mg: <0.1 (0.0-0.2)	Both doses: 0.1 (0.0-0.3)	15 mg: 0.3 (0.2-0.5); 30 mg: 0.5 (0.3-0.7)
Baricitinib AD [20]	IR: 0.19	DVT/PE IR: 0.07	IR: 0.39
Baricitinib AA [21]	IR: <0.1	VTE IR: 0.1	IR: 0.2
Abrocitinib AD consistent-dose age strata [22]	Not reproduced here; study reports overall and subgroup safety	Age ≥65: 1.7 (0.4-5.1); age 18 to <65: 0.1 (0.0-0.3)	Age ≥65: 2.4 (0.6-6.0); age 18 to <65: 0.1 (0.0-0.4)

Practical risk assessment and regulatory context

Clinical decisions should begin with the indication, disease severity, treatment history, and the prescribing information that applies to the selected product. A young patient without major risk factors is not automatically a candidate for first-line systemic JAK therapy. For example, the US indication for upadacitinib in AD specifies refractory moderate-to-severe disease that is inadequately controlled with other systemic treatments, including biologics, or situations in which those therapies are inadvisable. Treatment sequencing therefore needs to be considered within the specific indication [35].

Before starting systemic treatment, clinicians should review ASCVD, age, smoking history, hypertension, diabetes, dyslipidemia, obesity, prior VTE, and malignancy history. Other circumstances that may increase thrombotic risk, including planned surgery, immobility, estrogen exposure, and thrombophilia, may also influence treatment decisions. Active serious infection or clinically important laboratory abnormalities should be evaluated according to the selected agent's label. Baseline assessment commonly includes blood counts, hepatic function, lipids, infection screening, and vaccination status, although monitoring requirements vary by product [4,35].

For patients aged 65 years or older, current or long-term former smokers, and those at increased cardiovascular or cancer risk, the EMA recommends JAK inhibitors only when suitable alternatives are unavailable, with additional caution in patients at risk for VTE. In the United States, prescribing should follow the applicable FDA-approved label. Figure 2 summarizes these considerations in an author-proposed clinical workflow; it is intended as a practical aid rather than a validated prediction tool. The meaning of a suitable alternative also depends on the disease, since therapies used for AD are not necessarily appropriate alternatives for AA or PsA [3].

**Figure 2:**
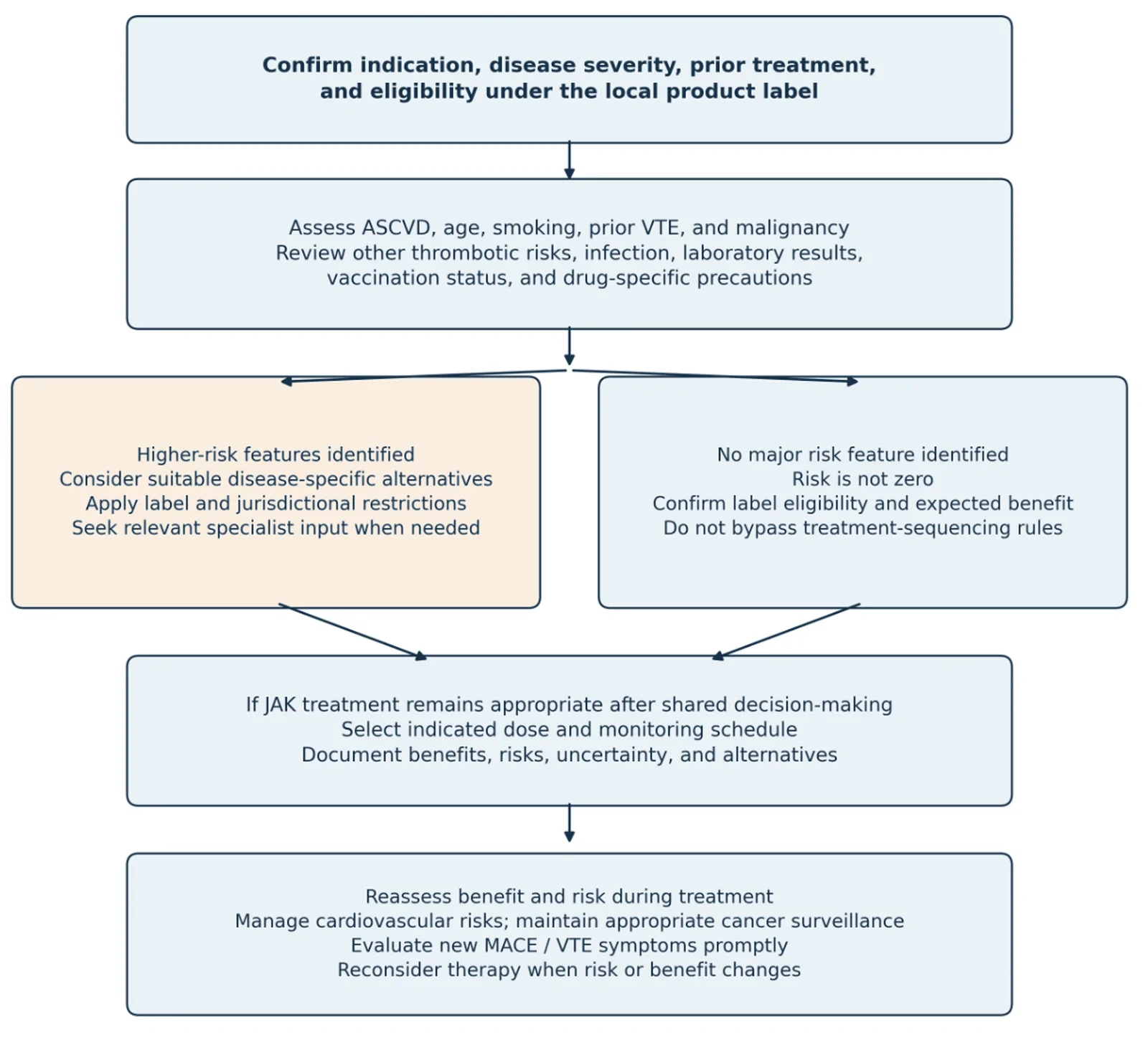
Assessment before systemic JAK treatment Author-proposed workflow for systemic JAK treatment. This is a qualitative aid, not a validated risk score or a substitute for indication-specific prescribing information. In the EMA framework, age ≥65 years, current or long-term previous smoking, or increased cardiovascular/cancer risk prompts use only when suitable alternatives are unavailable; VTE risk also warrants caution. Alternatives and monitoring vary by indication and jurisdiction [2-4,35]. ASCVD, atherosclerotic cardiovascular disease; EMA, European Medicines Agency; JAK, Janus kinase; MACE, major adverse cardiovascular event; VTE, venous thromboembolism.

When JAK treatment remains appropriate, the discussion with the patient should include expected benefits, uncertainty surrounding rare harms, available alternatives, and the need for continued reassessment. Clinicians should use the indicated dose and follow product-specific recommendations for dose reduction or the lowest effective dose when applicable. Appropriate cancer screening and skin surveillance should continue, modifiable cardiovascular risks should be addressed, and treatment should be reassessed when new diagnoses or changes in thrombotic risk occur. Routine laboratory testing cannot reliably predict MACE, VTE, or malignancy, so new symptoms suggestive of these events require prompt clinical evaluation rather than waiting for scheduled follow-up [2,4,35].

Limitations and future directions

This review has several limitations. The search strategy and selection of references introduce the possibility of selection bias. We did not apply a formal study-level risk-of-bias instrument or certainty-grading system, so the qualitative assessments presented here should not be interpreted as validated quality scores. Full reports were preferred when available, but some contextual findings were verified using abstracts and bibliographic records. This is particularly important when details about adjudication or subgroup methods were not fully accessible.

The underlying evidence has important limitations as well. Short controlled trials have limited power to detect uncommon outcomes. Long-term extension studies selectively retain patients who tolerate or benefit from treatment, and cumulative rates may obscure changes over time or depletion of susceptible patients. Trial eligibility criteria can underrepresent higher-risk patients, while maximum follow-up may give a misleading impression of typical exposure. Malignancy may also have a latency period longer than available follow-up. Many trials are industry-sponsored. Observational studies improve real-world applicability but introduce their own challenges, including confounding, exposure misclassification, and less consistent outcome ascertainment.

Future research should prioritize active-comparator studies within the same indication, transparent event adjudication, sufficient follow-up to evaluate malignancy, and stratification by clinically meaningful baseline risk. Analyses should distinguish between formulations, doses, and individual agents and should avoid double-counting patients from overlapping development programs. Independent registry-based surveillance and replication across different data sources will be particularly useful for determining whether observed differences reflect pharmacology, patient selection, prescribing patterns, or underlying patient characteristics.

## Conclusions

JAK inhibitors are valuable treatment options for immune-mediated diseases, but cardiovascular, thrombotic, and malignancy risks should be interpreted in the context of the individual patient rather than applied uniformly across indications. Age, smoking history, established cardiovascular disease, prior thromboembolism, and malignancy history should be assessed before treatment and incorporated into shared decision-making.

For dermatologists, the practical takeaway is that a JAK inhibitor should not be considered inherently high- or low-risk based solely on the drug class or dermatologic indication. Younger patients without major risk factors may have a more favorable benefit-risk profile, whereas older patients or those with significant cardiovascular, thrombotic, or malignancy risk warrant greater caution and consideration of alternative therapies. Treatment decisions should balance potential clinical benefit against patient-specific risk factors and available alternatives. Careful patient selection, appropriate monitoring, and periodic reassessment of risk are therefore central to the safe long-term use of JAK inhibitors. Continued long-term surveillance and well-designed comparative real-world studies will be necessary to determine whether individual JAK inhibitors differ in risk and to better identify patients who can receive these therapies with the most favorable long-term benefit-risk balance.
